# Comparative phylogeography of mainland and insular species of Neotropical molossid bats (*Molossus*)

**DOI:** 10.1002/ece3.5903

**Published:** 2019-12-19

**Authors:** Livia O. Loureiro, Mark D. Engstrom, Burton K. Lim

**Affiliations:** ^1^ Department of Natural History Royal Ontario Museum Toronto ON Canada; ^2^ Department of Ecology and Evolutionary Biology University of Toronto Toronto ON Canada

## Abstract

Historical events, habitat preferences, and geographic barriers might result in distinct genetic patterns in insular versus mainland populations. Comparison between these two biogeographic systems provides an opportunity to investigate the relative role of isolation in phylogeographic patterns and to elucidate the importance of evolution and demographic history in population structure. Herein, we use a genotype‐by‐sequencing approach (GBS) to explore population structure within three species of mastiff bats (*Molossus molossus*, *M. coibensis*, and *M. milleri*), which represent different ecological histories and geographical distributions in the genus. We tested the hypotheses that oceanic straits serve as barriers to dispersal in Caribbean bats and that isolated island populations are more likely to experience genetic drift and bottlenecks in comparison with highly connected ones, thus leading to different phylogeographic patterns. We show that population structures vary according to general habitat preferences, levels of population isolation, and historical fluctuations in climate. In our dataset, mainland geographic barriers played only a small role in isolation of lineages. However, oceanic straits posed a partial barrier to the dispersal for some populations within some species (*M. milleri*), but do not seem to disrupt gene flow in others (*M. molossus*). Lineages on distant islands undergo genetic bottlenecks more frequently than island lineages closer to the mainland, which have a greater exchange of haplotypes.

## Graphical abstract

In this manuscript, we use the genotype‐by‐sequencing approach on three species of Mastiff bats with different ecological histories and geographical distributions to explore population genetics parameters and better understand the role of geographic barriers in the dispersal and gene flow in bats. Our analyses show that the population structure of each species varies according to environmental factors, levels of population isolation, and demographic histories. We show that oceanic straights seem to pose a partial barrier for some species and that more isolated lineages on islands tend to undergo genetic bottlenecks more than connected lineages closer to the mainland.

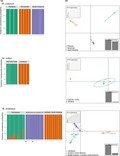

## 1 INTRODUCTION

Evolutionary geographic patterns and demographic histories of species are largely affected by historical events, habitat preferences, and geographic barriers (Bernatchez & Wilson, 1999; Querejeta & Castresana, 2018; Soltis, Morris, McLachlan, Manos, & Soltis, 2006). Moreover, several mechanisms might result in genetic isolation, such as geography and ecology, which often interact to shape the current pattern of diversity, but also making it difficult to distinguish the primary diversification factors (Wang & Bradburd, 2014). Populations in early stages of speciation provide good model systems to study these diversification mechanisms (Schield et al., 2015). Such groups have not yet developed secondary reproductive isolating mechanisms, and the primary genetic precursors of speciation are more easily discernable (Good, Dean, & Nachman, 2008; Orr, 1995).

Evolutionary processes might also act differently on insular and mainland populations, according to their degree of ecological and geographic isolation (Garcia‐Verdugo, Caujapé‐Castells, Mairal, & Monroy, 2018; Ortiz‐Ramírez, Sánchez‐González, Castellanos‐Morales, Ornelas, & Navarro‐Sigüenza, 2018). Populations on islands are more isolated, have a smaller meta‐population size, and are more vulnerable to habitat disturbance than mainland populations, which might alter insular population structure (Leisler & Winkler, 2015; Losos & Ricklefs, 2009; Spilani et al., 2019). Although alternative drivers of phylogeographic and population structure have been proposed for continental (Gray et al., 2019; Kalkvik, Stout, Hoffman, & Parkinson, 2018) and island populations (Čandek, Agnarsson, Binford, & Kuntner, 2018), few studies have explicitly compared the patterns between these two biogeographic systems (e.g., Pons et al., 2019). This comparison provides an opportunity to investigate the relative role of isolation, habitat selection, and demographic history in shaping phylogeographic patterns (Kalkvik et al., 2018; Sexton, Hangartner, & Hoffmann, 2014), especially using closely related species with similar life histories.

The Caribbean archipelago comprises numerous islands that differ in size, age, habitat, and level of isolation from other islands and from the mainland. In addition, this archipelago is located in proximity to two continents (North and South America), providing a varied landscape for testing phylogeographic hypotheses (Ricklefs and Bermingham, 2008). The Caribbean is divided into two main regions. The Lesser Antilles are located on the eastern margin of the Caribbean tectonic plate, and most of these islands were formed more than 20 Ma ago by volcanic activity and have never been connected (Bender et al., 1979; Donnelly, 1988; Pindell, 1994). In contrast, the Greater Antilles are much older and were formed during the separation of North and South America 170 Ma ago, with many islands remaining united until the Eocene (Iturralde‐Vinent & MacPhee, 1999; Kerr, Iturralde‐vinent, Saunders, Babbs, & Tarney, 1999; Pindell and Barrett, 1990). Pitman, Cande, Labrecque, and Pindell (1993) hypothesized that during dry periods in the Eocene, a land bridge formed connecting the Greater Antilles with Middle America, potentially resulting in faunal exchange between these landmasses. Iturralde‐Vinent and MacPhee (1999) also proposed that a short‐lived land bridge connected the Greater Antilles to northwestern South America during the Oligocene, promoting more recent faunal exchange.

Individual species and genera of bats are some of the few extant, native mammals in the Neotropics that occur on both the continental mainland and Caribbean islands. Bats are the second most speciose order of mammals and occupy almost all parts of the globe (Patterson, Willig, & Stevens, 2003), and their broad distribution renders them useful in comparative phylogeographic analyses. They are the only mammals capable of true flight and the dispersal ability of some groups allows these bats to colonize large geographic areas, including oceanic islands (Dávalos, 2007; Russell et al., 2016; Speer et al., 2017). Dispersal is essential to promote gene flow within a species, and the study of barriers that could isolate populations may provide important insights regarding how current and historical evolutionary processes effect speciation (Miller‐Butterworth, Jacobs, & Harley, 2003; Tam et al., 2005). Bats with high dispersal abilities usually exhibit little genetic structure among populations due to high rates of gene flow (Carstens, Sullivan, Davalos, Larsen, & Pedersen, 2004; McCracken, McCracken, & Vawter, 1994; Pumo, Goldin, Elliot, Phillips, & Genoways, 1988). These bats are potentially less affected by habitat disturbance and genetic fragmentation than more sedentary groups (Ibáñez, García‐Mudarra, Ruedi, Stadelmann, & Juste, 2006; Meyer, Kalko, & Kerth, 2009), although some exceptions have been reported in species of Molossidae inhabiting insular systems (Speer et al., 2017).

In the Caribbean, there are more than 60 species of bats (Dávalos, 2004, 2006; Loureiro, Gregorin, & Perini, 2018; Velazco & Patterson, 2013), but not much is known about the capacity of the different species to disperse among islands. Koopman (1977) suggested that oceanic straits present functional barriers for dispersal in bats in the Caribbean. Similarly, Genoways (1998) proposed that migration between islands was unlikely and predicted that gene flow among island populations was infrequent. Populations of bats are vulnerable to Caribbean hurricanes and volcanic eruptions, which may reduce population sizes and possibly result in accidental dispersal (Pedersen, 1998; Pedersen, Genoways, & Freeman, 1996). Therefore, even in the absence of regular inter‐island migration, the genetic diversification among some island populations could be muted by episodic gene flow. Likewise, populations from small and distant islands might be expected to be subjected to genetic drift more frequently than populations from large and less isolated islands, which can potentially decrease the genetic variability on small islands (Nei & Tajima, 1981). Previous studies have shown that although bats have significant capacity for dispersal, ocean straits may act as a barrier for some groups (Carstens et al., 2004; Fleming & Racey, 2013; García‐Mudarra, Ibáñez, & Juste., 2009; Larsen et al., 2012; Speer et al., 2017), but may not impose a strong barrier for others (Carstens et al., 2004; García‐Mudarra et al., 2009; Larsen et al., 2007; Pumo et al., 1988). This pattern might also have been affected by lower sea levels during the Pleistocene that shortened overwater distance, decreasing the oceanic barrier among some islands (Velazco & Patterson, 2013).

Mastiff bats of the genus *Molossus* represent an ideal model system for the study of population structuring on a broad geographic scale. *Molossus* are common aerial insectivores that inhabit a large range of habitats, from dry and humid semideciduous forests and tropical rainforests to pastures and savannas (Eger, 2008; Reid, 2009). Many species in this genus are well‐adapted to anthropogenic modifications, and they can be numerous in urban areas and degraded habitats (Taylor et al.,  2019). *Molossus* is nonmigratory, but many species are widely distributed and occur on both sides of prominent geographic barriers (e.g., Andes Mountains, Caribbean Sea) (Dolan, 1989; López‐González & Presley, 2001). Several species of *Molossus* are environmental generalists and are broadly distributed in the Neotropics, including on islands in the Caribbean. In contrast, other species in the genus are restricted to either mainland or Caribbean islands and prefer specific types of habitats, such as dry grasslands (Taylor et al.,  2019). The extensive distribution of *Molossus* throughout the Neotropics, including the Caribbean, suggests a strong colonizing ability and capacity to fly or to be carried by wind currents and storm systems over water. Although no studies have measured vagility in *Molossus*, other molossids have been reported to fly up to 160 km in a single night (McCracken et al., 2016), to reach speeds over 50 km/hr, to fly for up to 10 hr without resting (Marques, Rainho, Carapuço, Oliveira, & Palmeirim, 2004), and to migrate long distances (Cockrum, 1969; Glass 1958; Russell, Medellín, & Mccracken, 2005). Additionally, bats in the family Molossidae have relatively long, narrow wings with a reduced area, resulting in high wing loadings and high aspect ratios (Norberg & Rayner, 1987). This suite of adaptations is commonly associated with fast, long‐distance flight and enhanced dispersal abilities (Peterson, Eger, & Mitchell, 1995; Taylor et al., 2012; Burns & Broders, 2014).

Although phylogenetic relationships among some clades of *Molossus* are uncertain, 14 species are currently recognized (Lindsey & Ammerman, 2016; Lim, Loureiro, Upham, & Brocca, 2017; Loureiro, Gregorin, et al., 2018; Loureiro, Engstrom, & Lim, 2019; Loureiro et al., in review), but relationships among populations within species have not been examined. We investigated population structure in three species of *Molossus*, which represent the different ecological requirements and geographic distributions found in the genus: *M. coibensis*, *M. molossus*, and *M. milleri*. The most widespread species of *Molossus* is *M. molossus*, being present in both the mainland and Caribbean islands; *M. coibensis* is broadly distributed on the mainland, but is absent from the Caribbean, and *M. milleri* is the most broadly distributed species of the genus that is restricted to the Caribbean.

*Molossus coibensis* is one of the smallest species of *Molossus* and the second most widespread species of the genus, occurring from southern Mexico to southeastern Brazil (Dolan, 1989; Eger, 2008; Loureiro, Gregorin, et al., 2018; Reid, 2009). This species occurs in a variety of habitats, such as urban areas, evergreen forest, and dry and humid semideciduous forest, and it is only found in the mainland (Taylor et al., 2019).

*Molossus molossus* is larger than *M. coibensis* (Dolan, 1989) and is the most common and broadly distributed species of the genus, occurring from Argentina to the southern United States and the Lesser Antilles (Barquez, Mares, & Braun, 1999; Dolan, 1989; Eger, 2008; Fabian & Gregorin, 2007). This species also is present on both the west and east sides of the Andes*.* Several geographic populations were originally described as subspecies of *M. molossus* based on morphological characters but have been relegated as synonyms based on molecular and further morphological analyses (Dolan, 1989; Loureiro et al., 2019; Loureiro, Gregorin, et al., 2018). For instance, Loureiro et al. (in review), based on a molecular phylogeny, demonstrated that the subspecies *M. m. debilis* from Nevis and *M. m. pygmaeus* from Curacao group with other populations from South America within the *M. molossus* clade do not appear to be distinct lineages.

Several other supposed subspecies of *M. molossus* have been described that are confined to one or a few Caribbean islands (Dolan, 1989; Loureiro, Gregorin, et al., 2018). However, based on morphological characters, all Caribbean species and subspecies of *Molossus* were previously synonymized under the name *M. molossus* (Dolan, 1989; Eger, 2008; Simmons, 2005). Recent studies based on mitochondrial and nuclear genes, however, demonstrated that *M. verrilli* from the Dominican Republic, and *M. milleri* from Cuba, the Cayman Islands, and Jamaica are distinct species, restricting the distribution of *M. molossus* in the Caribbean to the Lesser Antilles and Puerto Rico (Lim et al., 2017; Loureiro et al., 2019; Loureiro, Gregorin, et al., 2018). *M. milleri* is morphologically similar to *M. verrilli* and *M. molossus* and occupies both forests and urban areas in the Greater Antilles (Taylor et al., in press).

Species within *Molossus* have low genetic variability, and mitochondrial and nuclear markers are often insufficient to resolve the relationship among some morphologically distinct species (Loureiro et al., 2019). However, the use of a next generation sequencing approach (NGS) has shown promise for resolving relationships and population structure in recently diversified groups in which the rate of genetic change is low (Cronin et al., 2015; Enk et al., 2016; Kusza et al., 2014; Lozier, 2014). One of these approaches is the genotyping‐by‐sequencing (GBS) method, which sequences many small tags in the genome flanking restriction sites. By this method, thousands of single nucleotide polymorphisms (SNPs) are recovered, vastly increasing the size of the overall dataset compared to typical Sanger methods. With this large dataset, consistent variance can be detected among genetically similar groups that are not revealed by standard gene sequencing approaches. In addition, genomic‐scale SNP data provide powerful options for testing patterns of genetic structure and demographic trends and to estimate population parameters for identifying changes in population size over time (Excoffier, Dupanloup, Huerta‐Sánchez, Sousa, & Foll, 2013; Gutenkunst, Hernandez, Williamson, & Bustamante, 2009; Lozier, 2014). These estimates may be important in a conservation context because they can indicate if a population has the potential to undergo inbreeding depression or has the genetic bandwidth to adapt to future environmental changes (Sovic, Carstens, & Gibbs, 2016).

Herein, we use the GBS approach on three species of Mastiff bats (*M*. *molossus*, *M. coibensis*, and *M. milleri*) with different ecological histories and geographical distributions to explore population genetic parameters and better understand the role of geographic barriers in dispersal and gene flow in bats. We tested the hypothesis proposed by Koopman (1977) and supported by Genoways (1998) that oceanic straits serve as barriers to dispersal by Caribbean bats. If this hypothesis is correct, we would expect significant genetic structuring and low gene flow among island populations and between islands and mainland populations when compared to levels of variation within island populations or within the mainland.

We also tested the hypothesis proposed by Nei and Tajima (1981) that more isolated populations are likely to experience genetic drift and bottlenecks relative to less isolated ones. To support this hypothesis, we would expect to find a decrease in the effective population size in island populations and a constant or increasing effective population size on the mainland. We would also expect to find that island populations with a mainland source are less affected by bottlenecks than insular populations isolated from other landmasses. As a result of different isolation patterns, we expect divergent phylogeographic structuring in mainland and insular species based on geographic barriers, habitat selection, and historical fluctuation in climate.

## 2 MATERIAL AND METHODS

### 2.1 Sample collection and library preparation

**Figure 1:**
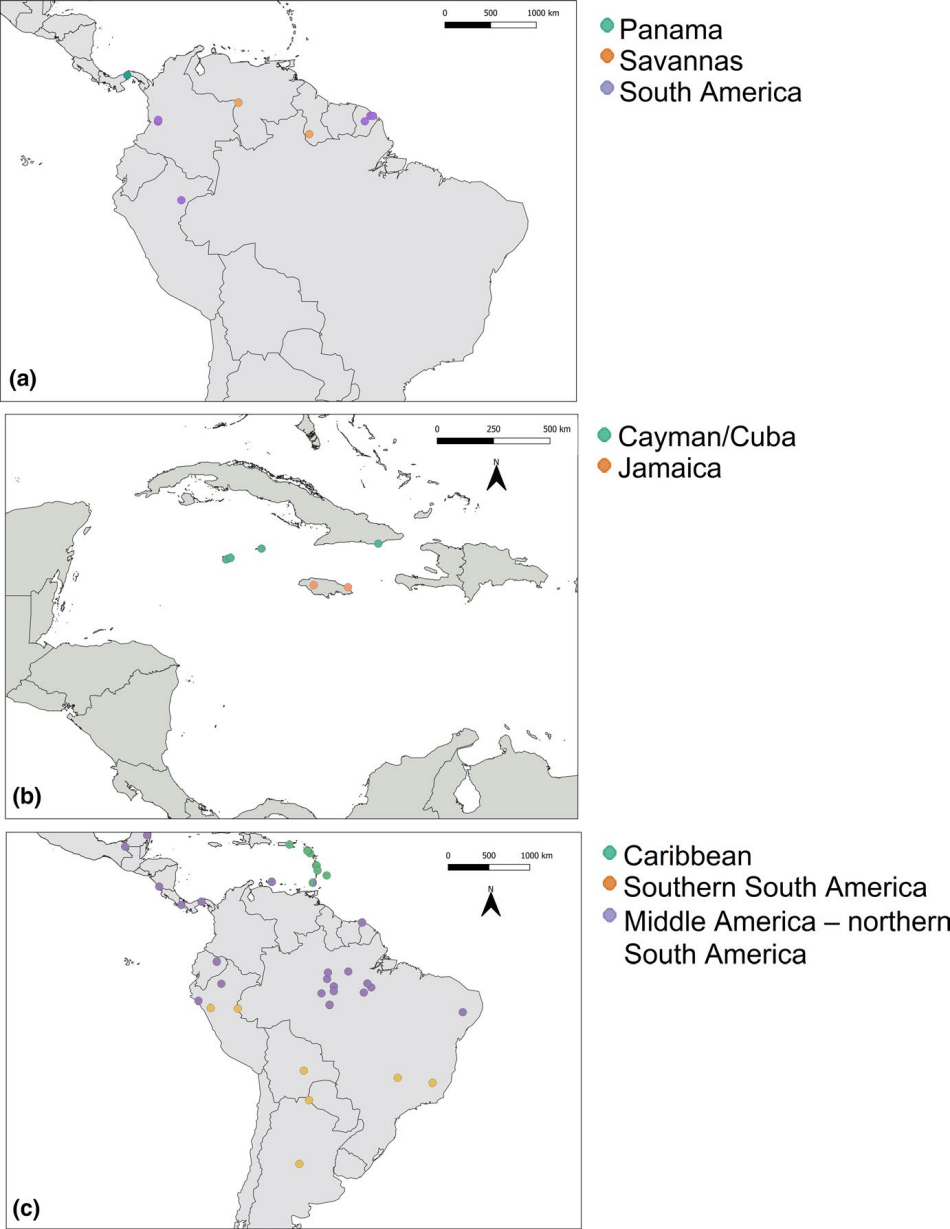
Maps of study areas and sampling location. (a) *M. coibensis*, (b) *M. milleri*, (c) *M. molossus*. Colors represent populations found in the structure and DAPC analyses

We obtained tissue samples from 62 *M. molossus* from South America, Middle America, and the Lesser Antilles, 20 *M. coibensis* from South and Middle America, and 19 *M. milleri* from the Greater Antilles (Figure 1; Appendix 1). Tissues samples included skeletal muscle, liver, heart, and kidney and were preserved in 95% ethanol or were frozen in liquid nitrogen upon collection of the specimen in the field. DNA extraction was conducted using Qiagen DNeasy extraction kit (Qiagen, Inc.) following standard protocols. Genomic DNA quality was checked by visual inspection on an agarose gel, and the DNA concentration was measured using a Nanodrop spectrophotometer (Nanodrop Technologies). We used 30 μl of DNA samples with concentrations higher than 100 ng/μl for library preparation and for the genotyping‐by‐sequencing approach following the protocol described by Elshire et al. (2011). All libraries were sequenced on an Illumina HiSeq 2000 in the Cornell Institute of Genomic Diversity (IGD).

### 2.2 Genotyping

De novo genotyping was performed using the Universal Network‐Enable Analysis Kit (UNEAK) pipeline, available on TASSEL 3.0 software (Bradbury et al., 2007). The sequences were trimmed to a 64 bp length, and shorter reads were discarded. In this pipeline, identical reads are clustered into tags and all unique tags are merged. No reference genome or GBS reference sequences of any species of *Molossus* are available. The phylogenetically closest genome available is from the family Vespertilionidae, which diverged from molossids in the Eocene (Lack, Roehrs, Stanley, Ruedi, & Ven Den Bussche, 2010; Soria‐Carrasco & Castresana, 2012; Teeling, Jones, & Rossiter, 2016). All three species were pooled and aligned for the reference genotyping before the dataset for each species was filtered and analyzed separately. Quality control and filtering of the reference genotypes of each species sample were also conducted on TASSEL 3.0 (Bradbury et al., 2007). Tags with depth lower than seven were treated as missing. The minimum allele frequency (MAF) value of 0.02 removed almost half of the SNPs from the original dataset of both the de novo and reference genotyping analyses, while any value above 0.02 had a very small impact on number of SNPs (Figure S1), which could suggest that these removed SNPs might represent informative rare alleles, rather than sequencing errors. The increase of the MAF value may cause an underestimation of heterozygotes because it may remove rare alleles, instead of the removal of sequencing errors, with the loss of biological information (Ni and Stoneking, 2016). Kim et al. (2011) argued that for rare SNPs (e.g., MAF < 0.01) it is not easy to differentiate between sequencing errors and a true rare allele, and alleles with less the 1% of MAF should be discarded. Linck and Battey (2019) showed that highly accurate population inferences are reached when relatively rare alleles are included (minimum allele count 2% to 8%). Therefore, in this study we set the MAF value at 2%.

We discarded individuals with more than 20% missing data. We also removed invariant SNPs and those with more than 10% missing data. The minimum heterozygosity proportion was set to 0.01. To remove linked sites in the alignment, SNPs <128 bp apart were removed. We tested for deviations of Hardy–Weinberg equilibrium in each dataset using TASSEL 3.0 (Bradbury et al., 2007). We estimated kinship between individuals within each species by exploring the relationship between identity by state (IBS), when two or more individuals share similar nucleotide sequences, using TASSEL 3.0 to ensure results were not conflicted by kinship (Rodríguez‐Ramilo & Wang, 2012).

### 2.3 Population structure analysis

We assigned individuals to populations under an admixture model with correlated allele frequencies using Structure v.2.3.4 (Pritchard, Stephens, & Donnelly, 2000). This model assumes that the common ancestor of all populations passed part of its genotype to all its descendants (Falush, Stephens, & Pritchard, 2003). The possible number of populations (*K*) in each species was estimated. To rule out population substructuring within samples of individuals for each species, we allowed *K* to range from 1 to a number in excess of the geographic locations. For example, we obtained samples of *M. milleri* from four different Caribbean islands, but we allowed *K* to vary between 1 and 10 (Figure S2). Five runs for each *K* were analyzed under a model of admixture and correlated allele frequency for 10 million generations each. The log likelihoods for each *K* value were averaged among runs and verified using log (Alpha) plots by interaction and ln* L* (*K*) by interaction. We used Structure Harvest v0.694 (Earl & vonHoldt, 2011) to assess the most likely number of populations, using the results of the Structure analyses. Patterns of individual assignment to clusters were also used to make an optimal inference regarding the *K* value for each species.

We assessed population differentiation of each species by conducting a principal component analysis (PCA) of pairwise individual genetic distances among populations and discriminant analysis of principal components (DAPC). We conducted these analyses converting the observed SNP data into principal components that summarize the variation between samples using the R package poppr (Kamvar, Tabima, & Grünwald, 2014). The relationships among clades within each species were investigated through a coalescence approach, which accounts for differences in genealogical histories of individual loci using the program SVDquartets (Chifman & Kubatkoin 2015) implemented in PAUP 4.0 (Swofford, 2003). Four independent runs were conducted to assess topological convergence, each including 500 bootstrap replicates and exhaustive quartet sampling. Trees were visualized using FigTree v. 1.4.3. We also generated a genetic distance tree to represent the genetic relatedness of the samples based on the UPGMA algorithm, with 500 bootstrap replicates. After the populations were defined, we visualized the posterior assignment of each sample using a composite stacked bar plot.

To quantify differentiation of allele frequency, we calculated the effective number of migrants (Nm) (Barton & Slatkinf, 1986) using Genepop 4.7.0 (Rousset, 2018). We also calculated the observed and expected heterozygosity, and the pairwise fixation index (Fst) between populations using Arlequin 3.5 (Excoffier & Lischer, 2010). The Fst was performed by calculating genetic distance based on all markers (after quality control and filtering) using a weighted analysis of variance (Cockerham, 1973; Weir & Cockerham, 1984). An analysis of molecular variance (AMOVA) for all pairs of populations was used to compute the divergence among populations using Arlequin 3.5.

### 2.4 Demographic inference

We generated joined population folded site frequency spectra (SFS) for each population within a species using Arlequin 3.5 (Excoffier & Lischer, 2010) with 100 bootstraps. To infer historical demography patterns in all populations, each SFS was imported into FastSimCoal26 (Excoffier et al., 2013) and the likelihood of three demographic models was compared using the likelihood ratio test (LRT) and Akaike's information criterion (AIC). The maximum log likelihood given the observed SFS was calculated to determine how well each model fit the data. The first model represents a constant population size, the second model represents a population expansion, and the third model imposes a bottleneck in each population. The last two models assume changes in population size and also calculate the present population size, the ancestral population size, and the time in number of generations since the growth or decline began.

All parameters used in the demographic models were selected from a uniform distribution, and we considered the mutation rate as 2.5 × 10^−8^ per nucleotide per generation. This value was calculated from human genomic data (Keightley, 2012; Nachman & Crowell, 2000) and is thought to provide a conservative estimate of population genetic parameters in mammals, including bats (Sovic et al., 2016). We calculated the confidence interval for each variable by performing a parametric bootstrap using point estimates from each parameter. For each model, we performed 100 runs (500,000 simulations per run) on FastSimCoal26 (Excoffier et al., 2013) and used the highest likelihood value in the LRT and AIC comparison. To ensure simulations were not stuck in a local maxima, individual runs of FastSimCoal26 under each model were tested for consistency and similar likelihood.

## 3 RESULTS

### 3.1 GBS data

A total of 436,086 SNPs were sequenced, and tags among individuals were aligned de novo. The species were then analyzed separately, and after initial filtering, the final GBS dataset with unlinked SNPs and only polymorphic sites consisted of 20 samples with 8,930 SNPs for *M. coibensis*, 54 samples with 7,311 SNPs for *M. molossus*, and 19 samples with 3,505 SNPs for *M. milleri*. Tests for Hardy–Weinberg equilibrium revealed significant linkage (*p* < .01) for seven pairs of loci in *M. molossus*, one pair of loci in *M. coibensis*, and no loci in *M. milleri*. One locus of each linked pair was removed to avoid bias in the results due to linked SNPs. Linkage disequilibrium is not common within subpopulations, except between very close or adjacent sites. In addition, isolated populations are less likely to exhibit strong correlations among loci (Pritchard et al., 2000). The initial removal of SNPs <128 bp apart likely removed almost all linked loci from our dataset, since only a few linked loci were found after the application of this filter. In addition, the low admixture among populations within all three study species might have also reduced linkage disequilibrium in our data. The IBS analysis did not reveal any closely related individuals in any of the three species, and no individuals were discarded (Figure S2).

### 3.2 Population structure analyses

**Figure 2:**
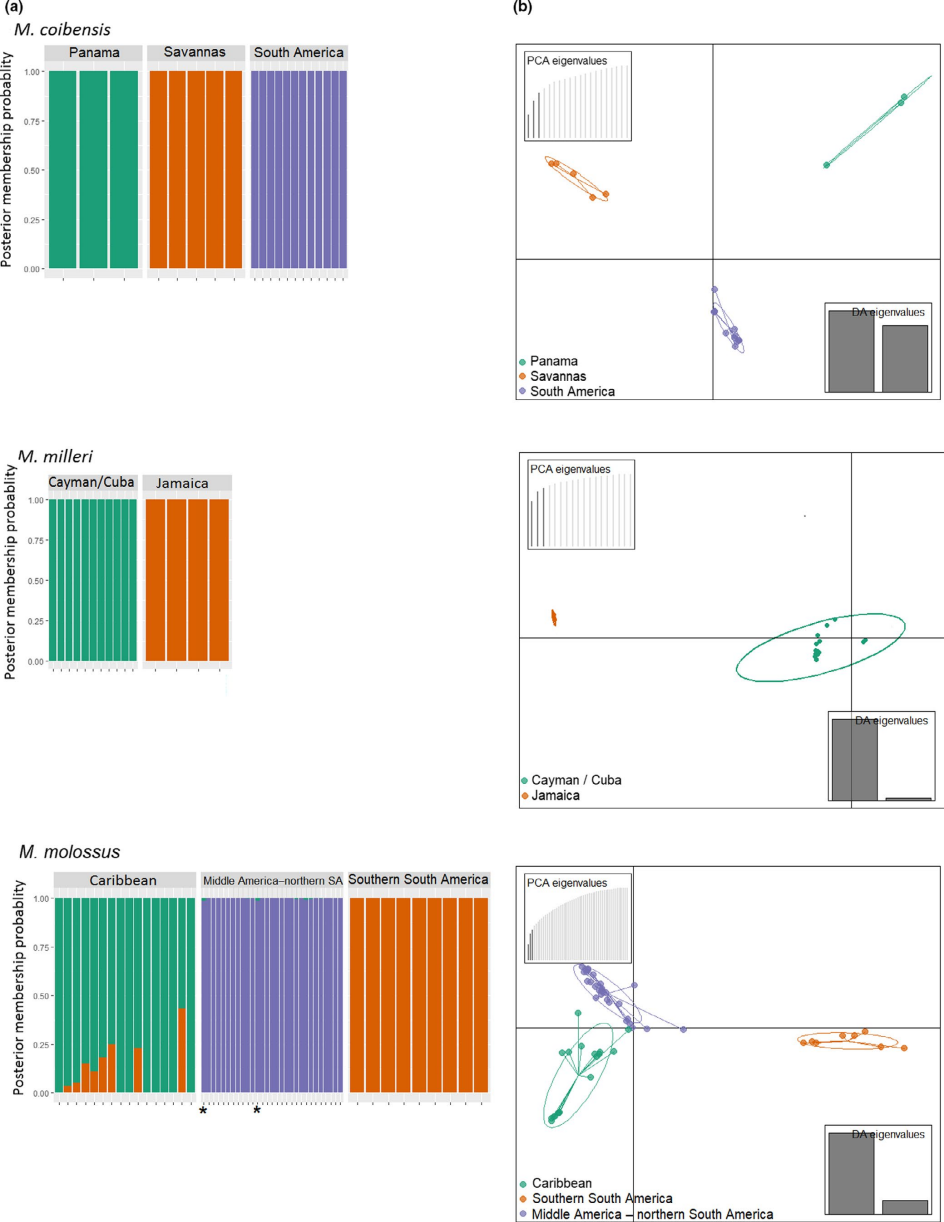
(a) Composite stacked bar plots under assumptions of *K* = 2 for *M. milleri* and *K* = 3 for *M. coibensis* and *M. molossus*. Each vertical bar along the *x*‐axis represents the genotype of an individual. Colors represent the haplotypes of each specimen. The *y*‐axis indicates the posterior probability of a genotype belonging to one or more clusters. * shows the individuals ROM125468 from Bonaire and TTU18551 from Grenada with migrant haplotypes. (b) Discriminant analysis of principal component (DAPC) with 95% confidence ellipses among populations within three species of *Molossus*: (a) *Molossus coibensis*, (b) *M. milleri*, and (c) *M. molossus*

The Structure analyses indicated a best fit of *K* = 3 for *M. coibensis*, *K* = 3 for *M. molossus*, and *K* = 2 for *M. milleri* (Figures 1 and 2a; Figure S3). Structure plots based on higher *K* values did not show any substructuring within populations (Figure S4). The composite stacked bar plots reveal that *M. coibensis* has three distinct populations: Panama, the savannas of Venezuela and Guyana, and rainforests of other South American countries (French Guiana, Ecuador, and Peru) (Figure 2a). *M. molossus* was also divided into three populations. The first population represents northern South America and Middle America with samples from northeast Brazil, Ecuador, French Guiana, Guyana, Mexico, Nicaragua, Panama, Bonaire, Grenada, and western Peru; the second population represents southern South America with individuals from Argentina, southeastern Brazil, Bolivia, Paraguay, and eastern Peru; and the third population comprises Caribbean samples from the Lesser Antilles and Puerto Rico. However, one of the four samples from Grenada (TTU18551) has almost 100% of its genotypes shared with the continental population and not with the Lesser Antilles (Figure 2a). The other three individuals from Grenada, as well as the samples from Martinique, had a higher probability of belonging to the Caribbean population, but they also share part of their genotypes with the Middle America–northern South America population. The samples from *M. milleri* were divided into two populations: one from Jamaica and one from the Cayman Islands and Cuba (Figure 2a).

The PCA and DAPC show similar patterns of pairwise genetic distance within each species (Figure 2b; Figures S5 and S6). The individuals of *M. coibensis* were separated into three main groups. The population from the savannas of Venezuela and Guyana appears more similar to Panama than to the other South American population in the PCA (Figure S5), but all three populations are equally distance in the DAPC space (Figure 2b). In *M. molossus*, samples were divided into three groups. The group from southern South America appears distinct, and the populations from Middle America–northern South America and from the Caribbean slightly overlap in the PCA but not in the DAPC (Figure 2b and Figure S5). In *M. milleri*, samples were divided into two distinct populations, one from Jamaica and the other from the Cayman Islands and Cuba (Figure 2b; Figure S5).

### 3.3 Phylogenetic relationships

**Figure 3:**
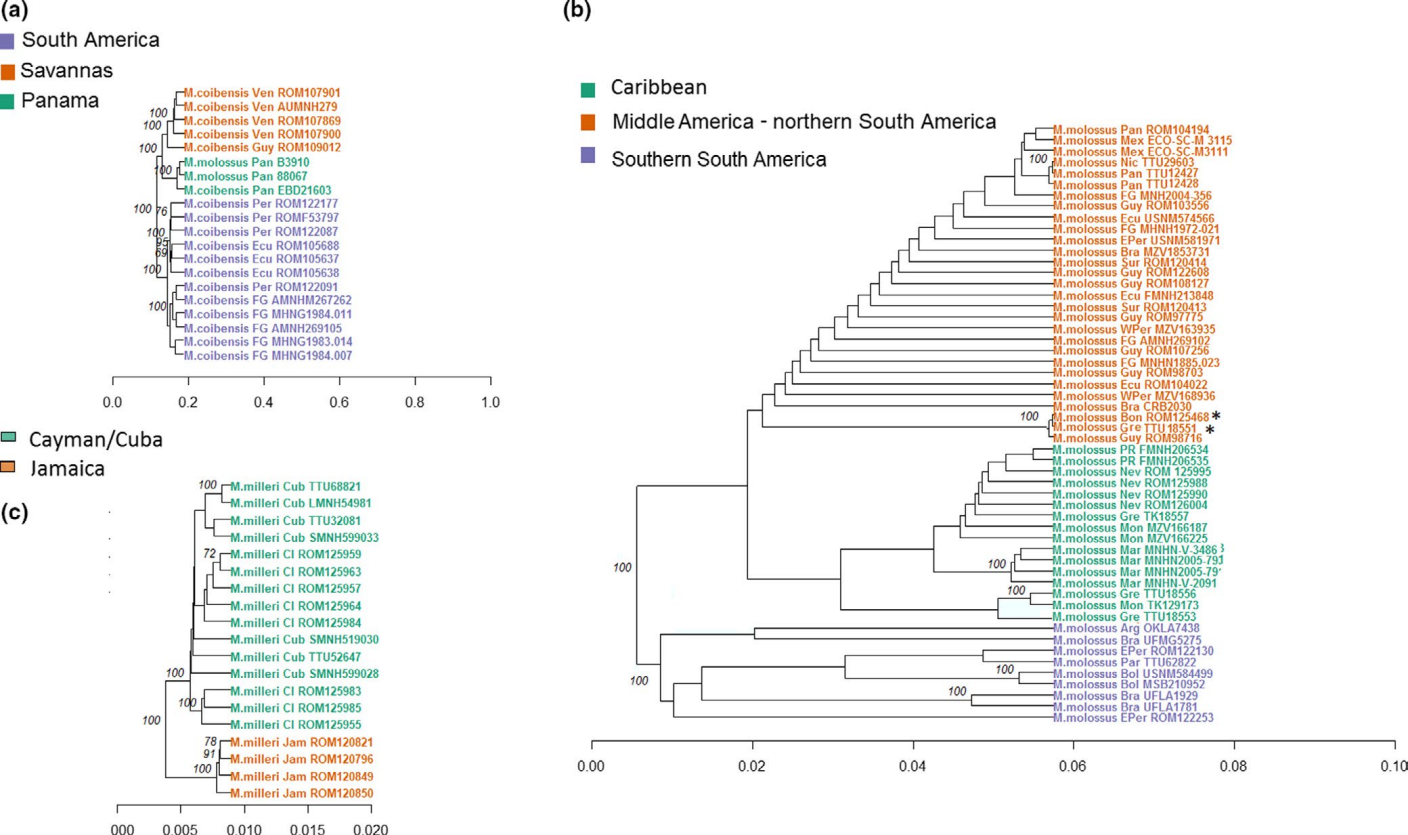
Distance trees of genetic similarity for three species of *Molossus*: (a) *M. coibensis*, (b) *M. molossus*, and (c) *M. milleri*. Nodes with greater than 90% bootstrap support are indicated. Colors represent population clusters, and * indicates samples where geographic location is different from population cluster

The phylogenetic and distance trees generated for each species also support the genetic structure found in the other analyses, although there were minor differences in relationships within each population between the analyses (Figure 3; Figure S7). In *M. coibensis*, individuals were primarily clustered by geographic location, and the three main groups had 100% bootstrap support (Figure 3a; Figure S7a). The individuals from savannas in Guyana and Venezuela cluster together and then group with those from Panama. In the third group, samples from rainforest in French Guiana cluster with those from Peru and Ecuador.

The phylogenetic and distance tree for *M. molossus* has three large clusters (Figure 3b; Figure S7b). The first group is composed of individuals from Middle America, northern South America, and from off‐shore islands (ROM125468 from Bonaire and TTU18551 from Grenada). The Caribbean group joined the Middle America–northern South America cluster, and grouped individuals from Puerto Rico and Lesser Antilles, including the other three samples from Grenada. The third group, comprising individuals from Argentina and southeastern Brazil, Paraguay, Bolivia, and western Peru, was an outlier to the two other groups. In the *M. milleri* trees, the Greater Antilles samples clustered into a Jamaica group and a Cayman + Cuba group (Figure 3c; Figure S7c).

### 3.4 Population diversity

The AMOVA results indicated significant genetic differentiation between each pair of populations within each analyzed species (*p* < .01; Table S1). Observed heterozygosity was lower than expected heterozygosity for all populations, except for *M. molossus* in the Middle America–northern South America population (Table 1). Designating a threshold of Fst > 0.2 as high population genetic differentiation (Frankham, Bradshaw, & Brook, 2014), pairwise comparisons of Fst between the southern South America and the Middle America–northern South America populations, and between the Caribbean and southern South America populations of *M. molossus* suggest some isolation (Table 1). Pairwise Fst was low between populations of *M. coibensis*, between the two populations of *M. milleri*, and between the Middle America–northern South America and the Caribbean populations of *M. molossus*, suggesting low levels of differentiation (Table 1). The estimated number of migrants was above one for all population pairs within species, except between *M. molossus* from southern South America and the Caribbean (0.699, Table 1).

**Table 1:** Quantitat parameters of population structure for three species of *Molossus*: *M. coibensis*, *M. molossus*, and *M. milleri*

Species	Fst	Nm	Population	Ho	He
*M. coibensis*	Panama × Savannas = 0.127	Panama × Savannas = 3.437	Panama	0.34 (0.24)	0.44 (0.11)
	Panama × SA = 0.180 Savannas × SA = 0.176	Panama × SA = 2.145 Savannas × SA = 2.341	Savannas	0.28 (0.20)	0.35 (0.14)
			SA	0.11 (0.22)	0.22 (0.11)
*M. molossus*	Middle America–northern SA × Caribbean = 0.122 Middle America–northern SA × Southern SA = 0.300 Caribbean × Southern SA = 0.417	Middle America–northern South America × Caribbean = 3.598 Middle America–northern SA × Southern SA = 1.167 Caribbean × Southern SA = 0.699	Middle America–northern SA	0.96 (0.05)	0.51 (0.02)
			Caribbean	0.29 (0.31)	0.36 (0.15)
			Southern SA	0.13 (0.15)	0.38 (0.13)
*M. milleri*	0.148	2.878	Jamaica	0.38 (0.34)	0.52 (0.15)
			Cuba/ Cayman Islands	0.50 (0.16)	0.85 (0.03)

Note

Numbers in parentheses represent the standard deviation of each value.

Abbreviations: Fst, pairwise fixation index; He, mean expected heterozygosity; Ho, mean observed heterozygosity; Nm, number of migrants between populations; SA, South America.

### 3.5 Demographic inferences

Likelihoods of each run in FastSimCoal under different demographic models were similar, indicating a convergence of the runs within a population and adequate searching of sample space. However, analyses suggest different demographic histories and qualitatively divergent parameter estimates among populations (Tables 2 and 3). The constant population size model was the best fit for *M. coibensis* from savannas and Panama and for *M. molossus* from southern South America. The South America population of *M. coibensis* and the Caribbean population of *M. molossus* had a higher likelihood value for the expansion model, but these values did not significantly differ from the constant model (Table 2). In contrast, the population expansion model was the best fit and significantly different from the constant model for *M. molossus* from the Middle America–northern South America population. The two groups of *M. milleri* had a different demographic history. For both populations of this species, the best fit model was the bottleneck, which was significantly different than the constant size model (Table 2).

**Table 2:** LRT and AIC for demographic models describing populations of three species of *Molossus*: *M. coibensis*, *M. molossus*, and *M. milleri*

Species	Population	Max ln *L*	Model	np	ln *L*	Null	LRT	*df*	*p*	AIC
*M. coibensis*	Panama		Constant	1	−1,418.02					10.3037
		−1,176.198	Expansion	3	−1,422.82	Constant	9.61	2	**.000**	10.3063
			Bottleneck	3	−1,542.16	Constant	248.27	2	**.000**	10.3997
*M. coibensis*	Savannas		Constant	1	−502.121					9.4016
		−354.110	Expansion	3	−503.690	Constant	3.14	2	**.043**	9.4065
			Bottleneck	3	−553.180	Constant	102.12	2	**.000**	9.4857
*M. coibensis*	South America		Constant	1	−156.000					8.3862
		−148.583	Expansion	3	−155.696	Constant	0.61	2	.738	8.3845
			Bottleneck	3	−167.388	Constant	22.78	2	**.000**	8.4474
*M. molossus*	Middle America–northern South America		Constant	1	−504.119					9.4051
		−498.010	Expansion	3	−500.981	Constant	6.28	2	**.043**	9.3996
			Bottleneck	3	−505.959	Constant	3.68	2	.159	9.4082
*M. molossus*	Caribbean		Constant	1	−501.756					9.4010
		−344.215	Expansion	3	−502.261	Constant	1.01	2	.604	9.4019
			Bottleneck	3	−548.154	Constant	92.80	2	**.000**	9.4778
*M. molossus*	Southern South America		Constant	1	−1,312.743					10.2364
		−1,269.769	Expansion	3	−1,326.488	Constant	6.24	2	**.000**	10.2454
			Bottleneck	3	−1,452.235	Constant	276.98	2	**.000**	10.3241
*M. milleri*	Cayman/Cuba		Constant	1	−28.577					
		−16.184	Expansion	3	−28.471	Constant	0.21	2	.899	6.9081
			Bottleneck	3	−25.088	Constant	6.98	2	**.031**	6.7989
*M. milleri*	Jamaica		Constant	1	−380.054					9.1569
		−349.914	Expansion	3	−380.848	Constant	1.59	2	.452	9.1594
			Bottleneck	3	−360.748	Constant	38.61	2	**.000**	9.1157

Note

Bold numbers indicate statistical significance (*p* < .05).

Abbreviations: *df*, degrees of freedom; ln *L*, ln likelihood; Max ln *L*, maximum log likelihood given the observed data; Np, number of parameters; Null, null model (constant model); *p*, probability that the bottleneck and expansion models are significantly different from the null model.

**Table 3:** Mean values and confidence intervals (CI) of present population size, ancestral population size, and the number of generations for observed population change for three species of *Molossus*: *M. coibensis*, *M. molossus*, and *M. milleri*

Species	Population		Population size	Ancestral population size	Number of generations
*M. coibensis*	Panama	Lower CI	65,360	65,360	0
		Mean	66,845	66,845	0
		Higher CI	67,617	67,617	0
*M. coibensis*	Savannas	Lower CI	59,037	59,037	0
		Mean	62,774	62,774	0
		Higher CI	65,327	65,327	0
*M. coibensis*	South America	Lower CI	65,739	65,739	0
		Mean	67,421	67,421	0
		Higher CI	69,216	69,216	0
*M. molossus*	Middle America–northern South America	Lower CI	67,883	49,033	67,981
		Mean	67,992	49,037	100,540
		Higher CI	68,158	49,495	123,170
*M. molossus*	Caribbean	Lower CI	44,249	44,249	0
		Mean	57,260	57,260	0
		Higher CI	69,301	69,301	0
*M. molossus*	Southern South America	Lower CI	63,466	63,466	0
		Mean	66,243	66,243	0
		Higher CI	70,550	70,550	0
*M. milleri*	Cayman/Cuba	Lower CI	14,916	65,978	163,714
		Mean	27,411	67,921	298,996
		Higher CI	42,250	69,169	442,312
*M. milleri*	Jamaica	Lower CI	3,155	65,241	148,549
		Mean	3,791	66,633	184,471
		Higher CI	4,578	68,013	206,784

Populations of *M. coibensis* appeared to have a constant effective population size through time (Table 3). The populations of *M. molossus* from southern South America and the Caribbean also did not appear to vary with time (Table 3). The best fit model assumes that the current population sizes of these groups did not change significantly over time and are the same as the ancestral population sizes. However, the *M. molossus* group from Middle America–northern South America increased about 14% in the last ~100 generations. The populations of *M. milleri* had the lowest current population size of any *Molossus*, especially for Jamaica. A bottleneck occurred earlier in the population from Cayman and Cuba in comparison with the group from Jamaica and had reduced population sizes of 40% and 56%, respectively (Table 3).

## 4 DISCUSSION

We examined three species of the mastiff bat (*Molossus*) with distinct geographic and ecological distribution patterns and found that their population structures have a low admixture of haplotypes and vary with habitat preferences, level of population isolation, and historical fluctuations in climate, resulting in mainland and insular species having different phylogeographic patterns. Mainland geographic barriers such as the Andes did not correspond to lineage breaks, except for the Amazon River, which acts as a filter barrier for *M. molossus* and also correlates with differences between rainforest and savanna habitats. Oceanic straits pose a partial barrier for some bats but not others, isolating populations of *M. milleri* between islands in the Greater Antilles, but with gene flow occurring among island populations of *M. molossus* within the Lesser Antilles. *M. molossus* populations from the mainland are distinct from those in the Lesser Antilles, indicating a degree of isolation. However, migrants from the continent found in the archipelago (such as Grenada) demonstrate ongoing gene flow between these two regions. For this group of bats, oceanic straits appear to act as a partial, filter barrier to dispersal. Our dataset also is consistent with the expectation that more isolated lineages on islands undergo genetic bottlenecks more frequently than lineages closer to the mainland.

### 4.1 Mainland populations

Geographic barriers in the Neotropics can disrupt dispersal in some birds (Weir, Faccio, Pulido‐Santacruz, Barrera‐Guzmán, & Aleixo, 2015; Weir & Price, 2011) and bats (Cuadrado‐Ríos & Mantilla‐Meluk, 2016; Dias, Santos Júnior, Perini, & Santos, 2017). The two species of bats examined in this study that occur in the mainland, *M. coibensis* and *M. molossus*, show little concordance between genetic structure and geological barriers. For example, both species fail to show phylogeographic structuring associated with the Andes or the Panamanian land bridge, and haplotypes are widespread across these potential geographic barriers. An exception is found within *M. molossus*, wherein two distinct groups are for the most part separated by the Amazon River, other than samples from Pernambuco, south of the Amazon River in northeastern Brazil, which group within the Middle America–northern South America population. The riverine barrier hypothesis proposes that rivers act as a barrier to gene flow, promoting divergence between populations on opposite banks (Wallace, 1852). The Amazon River in particular, which originated during the Miocene and attained its present course during the Pliocene, is thought to have contributed to allopatric speciation and population differentiation in many taxa (Baker et al., 2014; Nazareno, Dick, & Lohmann, 2017). Indeed, Amazonian rivers seem to be acting as dispersal barriers to several taxa of volant (Hayes & Sewlal, 2004) and nonvolant animals (Ayres & Clutton‐Brock, 1992; Bonvicino, Lindbergh, Faria, & Bezerra, 2012; Valdez & D'Elía, 2013). Conversely, rivers might not always act as barriers to gene flow in bats or small mammals. Many groups of bats have been reported to use smaller rivers as landmarks for orientation and migration pathways (Furmankiewicz & Kucharska, 2009; Serra‐Cobo, Lopez‐Roig, Marqués‐Bonet, & Lahuerta, 2000), and as preferred foraging habitats (Smith & Racey, 2008), suggesting that rivers might also act as dispersal corridors. In addition, the genetic patterns of some terrestrial small mammals do not corroborate the riverine hypothesis, with population substructure more pronounced along the length of the margins of the rivers, rather than between opposite sides (Patton & Da Silva, 1998; Patton, Silva, & Malcolm, 1994). In *M. molossus*, the Amazon River appears to act as a partial barrier to gene flow, despite the vagility of this bat, in accord with the riverine barrier hypothesis.

An additional explanation is that ecological factors also likely play an important role in population patterning. The division between these two populations of *M. molossus* is consistent with the transition between two large biomes in South America. The Middle America–northern South America population comprises individuals primarily distributed in tropical and subtropical rainforests, whereas the southern South America population inhabits mostly savannas, seasonal tropical forest, and agricultural landscapes. Exceptions are samples from eastern Peru, which occupy tropical forest. This distinction is supported by genetic differentiation based on the fixation index, whereby the southern South American population has higher Fst values in pairwise comparisons to the two other populations of *M. molossus*. Some groups of rodents and marsupials have a similar distribution and are restricted to each of these different habitats (Almeida, Bonvicino, & Cordeiro‐Estrela, 2007; Voss, 1991). Within each *M. molossus* lineage from the mainland, there is low intra‐population genetic divergence, a lack of obvious phylogeographic structure, and high levels of gene flow. This pattern is perhaps not surprising in a species with high dispersal abilities (Burns & Broders, 2014; Norberg & Rayner, 1987; Speer et al., 2017; Taylor et al., 2012).

*Molossus coibensis* was structured as three populations from: (a) tropical forests in Panama; (b) tropical forests of northern South America; and (c) savannas of Venezuela and Guyana. The Amazonian region of South America is composed primarily of rainforest, but patches of savanna are distributed from Venezuela to Suriname and southern Brazil (Haffer, 2002; Vanzolini & Williams, 1970). Repeated cycles of expansion and fragmentation of savannas in the Neotropics occurred most recently in the last 2 My, due to changes in temperature and sea level (Bennett, 1990), promoting opportunities for periodic connection and isolation between populations. Previous studies have reported the importance of the Amazonian savannas in the divergence of lineages of rodents (Bonvicino, Gonalves, Oliveira, Oliveira, & Mattevi, 2009), bats (Lim, 2010), lizards (Gainsbury & Colli, 2003), and birds (Naka, Cohn‐Haft, Mallet‐Rodrigues, Santos, & Torres, 2006). The specimens of the savanna population of *M. coibensis* were collected in the Llanos of Venezuela and Rupununi of Guyana, which are separated by a large region of rainforest (800 km) and by the Guiana Highland plateau (Lim & Lee, 2018). Although these geographic barriers could potentially decrease gene flow between these two savanna regions, other populations of different species of bats from the families Phyllostomidae, Vespertilionidae, Emballonuridae, and Molossidae are also united in phylogeographic analyses (Lim & Lee, 2018). These savanna regions were likely connected in the recent past (Sarmiento, 1983), and the distributional patterns observed today are the result of sundering of a common paleoenvironment rather than long‐distance dispersal across large expanses of Amazonian forest (Da Silva & Bates, 2002; Haffer, 1997; Sarmiento, 1983).

Previous studies based on mitochondrial and nuclear genes have reported that the population of *M coibensis* from the savannas formed a distinct clade relative to *M. coibensis* from forests of South and Middle America, suggesting that this population might belong to a putative new species (Lim & Engstrom, 2001; Lim & Lee, 2018; Loureiro et al., 2019). Although this population is genetically distinctive, it has a low degree of isolation with a high number of immigrants per generation (Table 1). Therefore, our data suggest that there is gene flow among these structured populations of *M. coibensis*, and the savanna population likely does not represent a distinct species.

### 4.2 Caribbean populations

Koopman (1977) and Genoways (1998) hypothesized that oceanic straits act as migration barriers in the Caribbean, which may result in low rates of gene flow among islands, and serve to isolate populations. In the Caribbean, *M. molossus* and *M. milleri* had distinct patterns of population differentiation, with evidence of oceanic straits acting as barriers to gene flow within *M. milleri* from the Greater Antilles, but not within *M. molossus* from the Lesser Antilles. The two populations of *M. milleri* are distinctive, but our analysis shows that there is some gene flow between Jamaica and Cayman/Cuba, and isolation is incomplete. Similarly, Muscarella, Murray, Ortt, Russell, and Fleming (2011), Carstens et al. (2004), and Larsen et al. (2012) found varying population structuring in different species of phyllostomid bats in the Caribbean, ranging from some species having monophyletic populations confined to individual islands, and other species lacking any genetic structuring among islands. Similar patterns have also been observed in other groups of volant animals, such as butterflies (Davies & Bermiham., 2001) and birds (Khimoun et al., 2016).

Two individuals from off‐shore islands (Bonaire and Grenada) had higher genetic similarity with the Middle America–northern South America group than to the Caribbean population. Bonaire is located approximately 80 km off the coast of Venezuela, and geologically considered a part of the continent, and Grenada is located about 160 km north of Trinidad, but geologically considered part of the Lesser Antilles. Due to the proximity of both islands to South America, they share similar fauna and flora with the mainland (Baker & Genoways, 1978), and these results are not unexpected, especially for Bonaire. However, some specimens from Grenada shared more haplotype similarity with other Lesser Antilles populations and clustered within the Caribbean group. These results indicate that there are two different haplotypes in Grenada, but because only one of our specimens from this island grouped within the Middle America–northern South America population, it suggests at least infrequent dispersal from the mainland (Pedersen et al., 1996). Speer et al. (2017) also reported mixed mainland/islands populations of the bat *Tadarida brasiliensis* in the Bahamas. The authors suggested that this pattern may have derived from independent colonization of the archipelago by divergent mainland lineages and that the genetic structure found is not due to isolation by oceanic barriers. Carstens et al. (2004) also reported distinct higher levels of genetic diversity among some populations of bats within individual Lesser Antilles islands, such as Montserrat, Nevis, and St. Kitts, and also suggested that this pattern could be derived from multiple colonization.

Some of the Caribbean islands are very small (e.g., Cayman Brac—19 km long), and in some islands, *Molossu*s was only caught in one or a few nearby geographic locations. Therefore, it was not possible to test for differences in gene flow among populations within individual islands compared with gene flow between different islands separated by similar geographic distance. However, in the islands where we could examine potential population differentiation, no population structuring was found within the same island, except for Grenada, which suggests lower genetic variation and higher gene flow within as compared to among different islands or between island and mainland populations.

### 4.3 Demographic histories

All mainland populations showed a constant or expanding effective population size through time, whereas the isolated Caribbean populations showed a higher probability of experiencing bottlenecks. These results support the hypothesis that island populations are more susceptible to bottlenecks than their continental relatives (Luikart, Sherwin, Steele, & Allendorf, 1998). Bottlenecks might cause heterozygosity deficiency in natural populations (Nei and Graur, 1984), decrease the genetic diversity of a population through random genetic drift (Groombridge, Jones, Bruford, & Nichols, 2000), reduce reproductive function (Madsen, Shine, Olsson, & Wittzell, 1999), and be involved in the speciation process (Mayr, 1963). Population persistence is highly connected to its evolutionary potential, which is enhanced by genetic variation (Frankel & Soulém, 1981; Newman & Pilson, 1997). Thus, level of genetic variance has direct implications for conservation management (Bouzat, 2010), especially in determining the minimum viable sizes of wild populations (Lande, 1988). These estimates may be important in a conservation context and can indicate if a population has the potential to undergo inbreeding depression or has the genetic breadth to adapt to future environmental changes (Sovic et al., 2016).

The recent divergence times associated with population expansion and bottlenecks among different lineages suggest that historical changes in climate during the Pleistocene affected the present phylogeographic patterns in these bats. *M*. *coibensis* showed no change in effective population size over time in its three populations. However, the other two species had a more complex demographic history. In *M. molossus* from the mainland, the lineage from southern South America showed no evidence of change in population size through time and the Middle America–northern South America population was a best fit expansion demographic model. Our analysis suggests that effective population size of the Middle America–northern South America lineage has increased about 14% over the last ~100k generations. Pacifici et al. (2013) estimated the generation length for *Molossus* as approximately 3.9 years. Using this estimation, *M. molossus* from Middle America–northern South America started to expand about 392 ka years ago. This period coincides with the middle Pleistocene, characterized by interglacial conditions possibly interrupted by climatic events associated with small‐scale glaciations (Rabassa and Clapperton, 1990). It has been hypothesized that a rise in global temperature 450 and 600 ka ago produced the longest and warmest interglacial episode during the Pleistocene (Verzi, Deschamps, & Tonni, 2004), which resulted in a distributional expansion of several species (Kozma, Melsted, Magnusson, & Hoglund, 2016; Lambeck, Yokoyama, & Purcell, 2002; Martizez‐Freiria, Velo‐Anton, & Brito, 2015; Vrba, 1985).

Species in the Caribbean showed contrasting patterns of demographic history. Both populations of *M. milleri* (from Jamaica, and Cuba and Cayman Islands) closely fit the bottleneck model, and the population of *M. molossus* from the Lesser Antilles showed a higher likelihood for the constant population size model. Considering the same generation length of 3.9 years estimated for *M. molossus* and for other species of the genus (Pacifici et al., 2013), the effective population size decline in *M. milleri* started around 1.1 mya in the group from Cuba and the Cayman Islands, and about 719 ka in the group from Jamaica. These dates correspond to the early and beginning of the middle Pleistocene, respectively, which was characterized by several cycles of glacial and interglacial climates (Raymo, Ganley, Carter, Oppo, & McManus, 1998). These cycles likely caused fluctuations in temperature and sea level, resulting in the extinction of some Caribbean bat species during the late Pleistocene (Dávalos & Russell, 2012; Morgan, 2001). Therefore, we hypothesize that climate change in the early Pleistocene likely generated ecological changes and repetitive fluctuations in size of island landmasses, affecting population sizes of these bats. However, the confidence intervals of ancestral and extant effective population sizes overlapped within the two populations of *M. milleri* and caution is needed in the interpretation of these data. Two alternative scenarios, the anthropogenic colonization during the Quaternary (Valente, Etienne, & Dávalos, 2017) and the Holocene last glacial–interglacial transition (Soto‐Centeno & Steadman, 2015), have also been proposed to explain the extinction of bats in the Caribbean and show that fluctuations in population sizes might be more complex than previously thought.

The differences in demographic history among Caribbean lineages of *Molossus* might be explained by historical biogeography and current degree of isolation. *M. milleri* is restricted to the Greater Antilles, with no apparent connection to the mainland. In contrast, *M. molossus* from the Lesser Antilles receives migrants from northern South America, and mainland and island populations exhibit low levels of population differentiation. For example, the two specimens from Bonaire and Grenada with mainland haplotypes are indicative of gene flow between the areas. This haplotype exchange might have resulted from accidental and isolated events or indicates that the continent is acting as a source population of migrants to the Caribbean population, in particular for Bonaire because of its proximity to Venezuela. Populations that are isolated on islands might decrease drastically in size due to environmental changes and catastrophic events (Pedersen, 1998; Pedersen et al., 1996), and genetic drift in these small populations may result in the loss or decrease of some allele frequencies (Keller et al., 2001). Increased levels of immigration can lead to very different genetic outcomes from those expected in isolated populations. Island lineages with immigration sources can recover much faster from a bottleneck than isolated populations, despite increases in average inbreeding on islands (Keller et al., 2001).

Buerlke and Gompert (2012) found that population allele frequencies were more variable for smaller samples of individuals (2–10), which could potentially affect results in both populations structuring and demographic analyses. However, Pluzhnikov and Donnelly (1996) and Beerli (2004) argue that the effect of small sample size on the estimates of population structuring and gene flow is minimum, except that the confidence intervals are somewhat larger with fewer individuals. Nadeau et al. (2011) proposed that a deeper genetic coverage could ameliorate the effect of a small number of individuals. In addition, other studies with next generation sequencing have conducted similar population genetic and demographic analyses using different population sizes (from 5 to 27 individuals) and found consistent results (Sovic, Fries, Martin, & Gibbs, 2018). Robison, Coffman, Hickerson, and Gutenkunst (2014) also found accurate parameter and demographic estimates for populations with more ancient demographic events (in the order of 0.5*N*e_ _generations ago) in small numbers of sampled individuals. In our data, when the models that included changes in population size had a higher likelihood, all the events (bottlenecks or expansions) occurred more than 0.5Ne generations ago, corroborating with Robison et al. (2014).

Our study of phylogeographic patterns in mainland and island populations in a group of highly mobile bats (*Molossus*) found that the Amazon River and ecological habitats (rainforest and savanna), but not the Andes Mountains, have an effect on the genetic structuring of *M. molossus* in South America. By contrast, oceanic barriers in the Greater Antilles play a role in isolation of some species (*M. milleri*), and that these isolated populations are more subject to bottlenecks and therefore vulnerable to environmental change. We expect these patterns to be even more pronounced in populations of bats with lower dispersal abilities, such as fruit and nectar feeding phyllostomids. Demographic research on the bat fauna as a whole would provide important information relevant to biological conservation in the Caribbean as climate change and environment vulnerability accelerate.

## CONFLICT OF INTEREST

None declared.

## AUTHORS CONTRIBUTION

L. Loureiro, M. Engstrom, and B. Lim collected the data through field trips in several Neotropics countries. L. Loureiro conducted sequencing and analyses. M. Engstrom and B. Lim verified the analytical methods and supervised the findings of this work. All authors contributed to the interpretation of the results. L. Loureiro wrote the manuscript with support from M. E. and B. Lim.

## Data Availability

VCF files with SNP data are available at https://doi.org/10.5061/dryad.d7wm37pxc

## APPENDIX 1

### SPECIMEN VOUCHERS, SPECIES IDENTIFICATION, COUNTRY OF ORIGIN, LOCALITY, AND COORDINATES FOR *MOLOSSUS* USED IN THE GENETIC ANALYSES

**Table:**

Sample name	Genus	Species	Country	Locality	Latitude	Longitude
ROM 105,638	*Molossus*	*coibensis*	Ecuador	Napo	39.000	−76.270
ROM 105,637	*Molossus*	*coibensis*	Ecuador	Napo	39.000	−76.270
ROM 105,688	*Molossus*	*coibensis*	Ecuador	Napo	3.900	−76.270
MHNG 19,884.011	*Molossus*	*coibensis*	French Guiana	Cacao	4.568	−52.468
MNHG 19,883.014	*Molossus*	*coibensis*	French Guiana	Cacao	4.568	−52.468
MHNG 1984.007	*Molossus*	*coibensis*	French Guiana	Kaw	4.550	−52.167
AMNH 267,262	*Molossus*	*coibensis*	French Guiana	Cayenne	3.9332	−53.088
AMNH 269,105	*Molossus*	*coibensis*	French Guiana	Cayenne	3.9332	−53.088
ROM 119,012	*Molossus*	*coibensis*	Guyana	Dadanawa Ranch	2.4927	−59.313
ROM 53,797	*Molossus*	*coibensis*	Peru	Loreto	4.899	−73.650
PAN 88,067	*Molossus*	*coibensis*	Panama	Gamboa	9.116	−79.699
PAN B3910	*Molossus*	*coibensis*	Panama	Gamboa	9.116	−79.699
EBD 21,603	*Molossus*	*coibensis*	Panama	Gamboa	9.116	−79.699
ROM 122,087	*Molossus*	*coibensis*	Peru	Loreto	4.899	−73.650
ROM 122,091	*Molossus*	*coibensis*	Peru	Loreto	4.899	−73.650
ROM 122,177	*Molossus*	*coibensis*	Peru	Loreto	4.899	−73.650
ROM 107,901	*Molossus*	*coibensis*	Venezuela	Amazonas	6.030	−67.250
ROM 107,900	*Molossus*	*coibensis*	Venezuela	Amazonas	6.030	−67.250
ROM 107,869	*Molossus*	*coibensis*	Venezuela	Amazonas	6.030	−67.250
AUMNH 279	*Molossus*	*coibensis*	Venezuela	Amazonas	6.030	−67.250
ROM 125,985	*Molossus*	*milleri*	Cayman Islands	Cayman Brac	19.699	−79.856
ROM 125,983	*Molossus*	*milleri*	Cayman Islands	Cayman Brac	19.699	−79.856
ROM 125,955	*Molossus*	*milleri*	Cayman Islands	Grand Cayman	19.314326	−81.169
ROM 125,963	*Molossus*	*milleri*	Cayman Islands	Grand Cayman	19.332	−81.104
125,957 ROM	*Molossus*	*milleri*	Cayman Islands	Grand Cayman	19.314	−81.169
ROM 125,959	*Molossus*	*milleri*	Cayman Islands	Grand Cayman	19.271	−81.281
ROM 125,984	*Molossus*	*milleri*	Cayman Islands	Cayman Brac	19.699	−79.856
ROM 125,964	*Molossus*	*milleri*	Cayman Islands	Grand Cayman	19.332	−81.104
SMNH 59,028	*Molossus*	*milleri*	Cuba	Guantanamo Bay	19.900	−75.150
SMNH 519,030	*Molossus*	*milleri*	Cuba	Guantanamo Bay	19.900	−75.150
SMNH 599,033	*Molossus*	*milleri*	Cuba	Guantanamo Bay	19.900	−75.150
LMNH 54,981	*Molossus*	*milleri*	Cuba	Guantanamo Bay	19.900	−75.150
TTU 52,647	*Molossus*	*milleri*	Cuba	Guantanamo Bay	19.900	−75.150
TTU 32,081	*Molossus*	*milleri*	Cuba	Guantanamo Bay	19.900	−75.150
TTU 68,821	*Molossus*	*milleri*	Cuba	Guantanamo Bay	19.900	−75.150
TTU 22,195	*Molossus*	*milleri*	Jamaica	St. Ann	18.436	−77.201
ROM 120,849	*Molossus*	*milleri*	Jamaica	Portland	18.14347	−76.373
ROM 120,796	*Molossus*	*milleri*	Jamaica	Saint Elizabeth	18.227	77.75446
ROM 120,821	*Molossus*	*milleri*	Jamaica	Portland	18.14347	−76.373
ROM 120850	*Molossus*	*milleri*	Jamaica	Portland	18.14347	−76.373
ROM 120798	*Molossus*	*milleri*	Jamaica	Saint Elizabeth	18.227	77.75446
OKLA 7,438	*Molossus*	*molossus*	Argentina	Jujuy	−32.600	−63.883
TTU 151411	*Molossus*	*molossus*	Barbados	St Thomas Parish	13.194	−59.543
USNM 584,499	*Molossus*	*molossus*	Bolivia	Santa Cruz	−17.786	−63.181
MSB 210,952	*Molossus*	*molossus*	Bolivia	Santa Cruz	−17.786	−63.181
ROM 125,468	*Molossus*	*molossus*	Bonaire	Playa Frans	12.202	−68.262
MZV 1,853,731	*Molossus*	*molossus*	Brazil	Pernambuco	−8.472	−37.947
UFLA 1781	*Molossus*	*molossus*	Brazil	Minas Gerais	−19.717	−42.750
CRB 2030	*Molossus*	*molossus*	Brazil	Not provided	Not provided	Not provided
UFLA 1929	*Molossus*	*molossus*	Brazil	Minas Gerais	−19.717	−42.750
UFMG 5,275	*Molossus*	*molossus*	Brazil	Minas Gerais	−18.919	−48.277
ROM 104,022	*Molossus*	*molossus*	Ecuador	Napo	−3.990	−76.270
FMNH 213,848	*Molossus*	*molossus*	Ecuador	Tiguino	−0.462	−76.993
USNM 574,566	*Molossus*	*molossus*	Ecuador	Orellana	−0.466	−76.987
AMNH 269,102	*Molossus*	*molossus*	French Guiana	Cayenne	−3.933	−53.088
MHNG 1885.023	*Molossus*	*molossus*	French Guiana	Awala‐Yalimapo	−5.7411	−53.928
MHNH 2004‐356	*Molossus*	*molossus*	French Guiana	Angoulème	−5.400	−53.650
MHNG 1972‐021	*Molossus*	*molossus*	French Guiana	Cacao	−4.568	−52.468
TTU 18,551	*Molossus*	*molossus*	Grenada	St George	12.056	−61.748
TTU 18,553	*Molossus*	*molossus*	Grenada	St George	12.056	−61.748
TTU 18,556	*Molossus*	*molossus*	Grenada	St George	12.056	−61.748
TTU 18,557	*Molossus*	*molossus*	Grenada	St George	12.056	−61.748
ROM 97,775	*Molossus*	*molossus*	Guyana	Upper Takutu‐Upper Essequibo	−3.230	−59.480
ROM 98,716	*Molossus*	*molossus*	Guyana	Barima‐Waini	−7.340	−59.090
ROM 122,608	*Molossus*	*molossus*	Guyana	Upper Takutu‐Upper Essequibo	−2.182	−59.337
ROM 107,256	*Molossus*	*molossus*	Guyana	Potaro‐Siparuni	−4.400	−58.410
ROM 103,556	*Molossus*	*molossus*	Guyana	Upper Demerara‐Berbice	−5.180	−58.420
ROM 98,703	*Molossus*	*molossus*	Guyana	Barima‐Waini	−7.340	−59.090
ROM 108,127	*Molossus*	*molossus*	Guyana	Cuyuni‐Mazaruni	−5.520	−60.370
MHNH 2005‐791	*Molossus*	*molossus*	Martinique	Le Precheur	14.800	−61.217
MHNH 2055‐793	*Molossus*	*molossus*	Martinique	Le Precheur	14.800	−61.217
MHNH V‐2091	*Molossus*	*molossus*	Martinique	Morne Rouge	14.767	−61.133
MNHN/V3486	*Molossus*	*molossus*	Martinique	Morne Rouge	14.767	−61.133
ECO‐SC‐M 3,111	*Molossus*	*molossus*	Mexico	Tabasco	17.800	−91.533
ECO‐SC‐M 3,115	*Molossus*	*molossus*	Mexico	Tabasco	17.800	−91.533
ECO‐SC‐M 5675	*Molossus*	*molossus*	Mexico	Quintana Roo	19.578	−88.045
MZV 166,187	*Molossus*	*molossus*	Montserrat	Belham River	16.749	−62.193
MZV 166,225	*Molossus*	*molossus*	Montserrat	Belham River	16.749	−62.193
TTU 129,173	*Molossus*	*molossus*	Montserrat	Belham River	16.749	−62.193
TTU 151275	*Molossus*	*molossus*	St. Lucia	Castries	13.996	−61.006
ROM 125,988	*Molossus*	*molossus*	Nevis	Newcastle	17.191	−62.586
ROM 125,990	*Molossus*	*molossus*	Nevis	Newcastle	17.196	−62.596
ROM 125,995	*Molossus*	*molossus*	Nevis	Newcastle	17.196	−62.596
ROM 126,004	*Molossus*	*molossus*	Nevis	Pond Hill	17.124	−62.593
TTU 29,603	*Molossus*	*molossus*	Nicaragua	Rivas	11.470	−86.125
ROM 104,194	*Molossus*	*molossus*	Panama	Gamboa	9.060	−79.420
TTU 12,427	*Molossus*	*molossus*	Panama	Chiriqui	8.517	−82.617
TTU 12,428	*Molossus*	*molossus*	Panama	Chiriqui	8.517	−82.617
TTU 62,822	*Molossus*	*molossus*	Paraguay	Boqueron	−22.452	−62.348
TTU 129172	*Molossus*	*molossus*	Paraguay	Boqueron	−22.452	−62.348
ROM 122,253	*Molossus*	*molossus*	Peru	Loreto	−9.899	−73.650
MZV 163,935	*Molossus*	*molossus*	Peru	Loreto	−9.899	−73.650
ROM 122256	*Molossus*	*molossus*	Peru	Loreto	−9.899	−73.650
MZV 168,936	*Molossus*	*molossus*	Peru	Lambayerque	−6.700	−79.900
LMNH 7545	*Molossus*	*molossus*	Peru	Amazonas	−9.825	−77.948
USNM 581,971	*Molossus*	*molossus*	Peru	Cordillera Del Condor	−4.095	−78.393
ROM 122,131	*Molossus*	*molossus*	Peru	Loreto	−9.899	−73.650
FMNH 206,534	*Molossus*	*molossus*	Puerto Rico	Vieques Island	18.125	−65.442
FMNH 206,535	*Molossus*	*molossus*	Puerto Rico	Vieques Island	18.125	−65.442
TTU 151296	*Molossus*	*molossus*	St. Lucia	Castries	13.996	−61.006
ROM 120,414	*Molossus*	*molossus*	Suriname	Sipaliwini	−2.026	−56.124
ROM 120,413	*Molossus*	*molossus*	Suriname	Sipaliwini	−2.026	−56.124

Samples that were excluded due to high missing data.
