# Supplementary material for: Comparative phylogeography of mainland and insular species of Neotropical molossid bats (Molossus)
Source: Ecol Evol. 2019 Dec 19;10(1):389–409. doi: 10.1002/ece3.5903 (PMC6972955; doi:10.1002/ece3.5903)
Supplement: Supplementary file 1 [file ECE3-10-389-s001.docx]

## *Ecology and Evolution* Supporting Information

Article title: Comparative phylogeography of mainland and insular species of Neotropical molossid bats (*Molossus*)

Authors: Loureiro, L.O., Engstrom, M.D., Lim, B.K.

The following Supporting Information is available for this article:

Table S1 – AMOVA values for each population pair within three species of *Molossus*

| Species | Populations | P value |
| --- | --- | --- |
|  | Panama x Savannas | <0.001 |
| *M. coibensis* | Panama x SA  Savannas x SA | <0.001 |
|  |  | <0.001 |
| *M. molossus* | Middle America-northern SA x Caribbean  Middle America - northern SA x Southern SA  Caribbean x Southern SA | 0.005 |
|  |  | 0.002 |
|  |  | <0.001 |
| *M. milleri* | Cayman/Cuba x Jamaica |  |
|  |  | <0.001 |

Figure S1 – Effect of minimum allele frequency values on the number of SNPs removed in the dataset. Note that for values higher than 0.02 thare is not a significant decrease in number of SNPs.

Figure S2 – Kinship graphs showing identity by state (IBS) values for all pairs of individuals for three species of *Molossus*. Diagonal squares represent the maximum IBS for each individual, and differences in colors represent deviations from these values. Note that no pair of individuals shows high IBS value, indicating a lack of kinship among pairs.


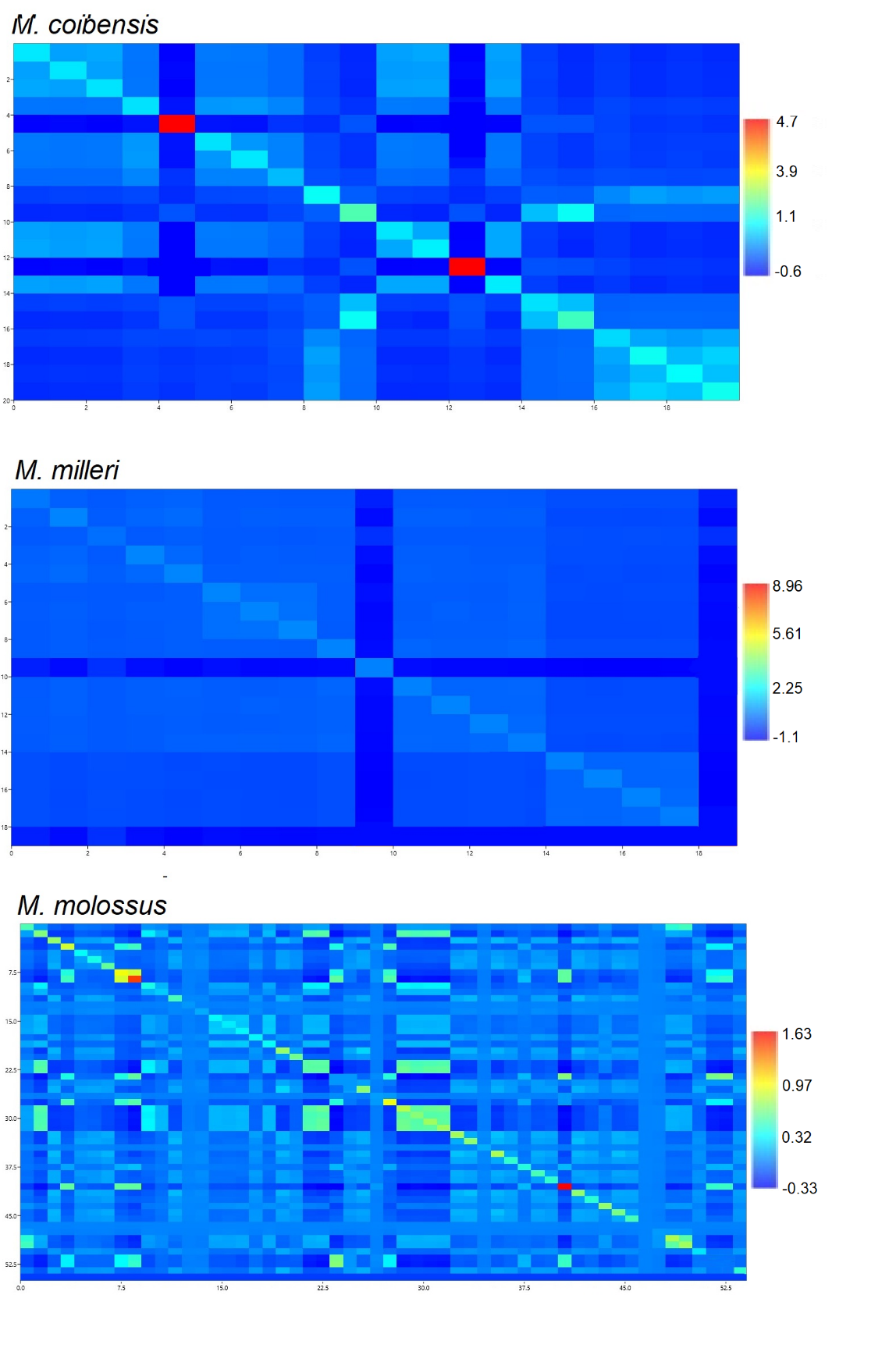


Figure S3 - Genetic clustering results from STRUCTURE for 3 species of *Molossus*. The mean −lnL is shown for each value of k, where k is the number of clusters assumed.


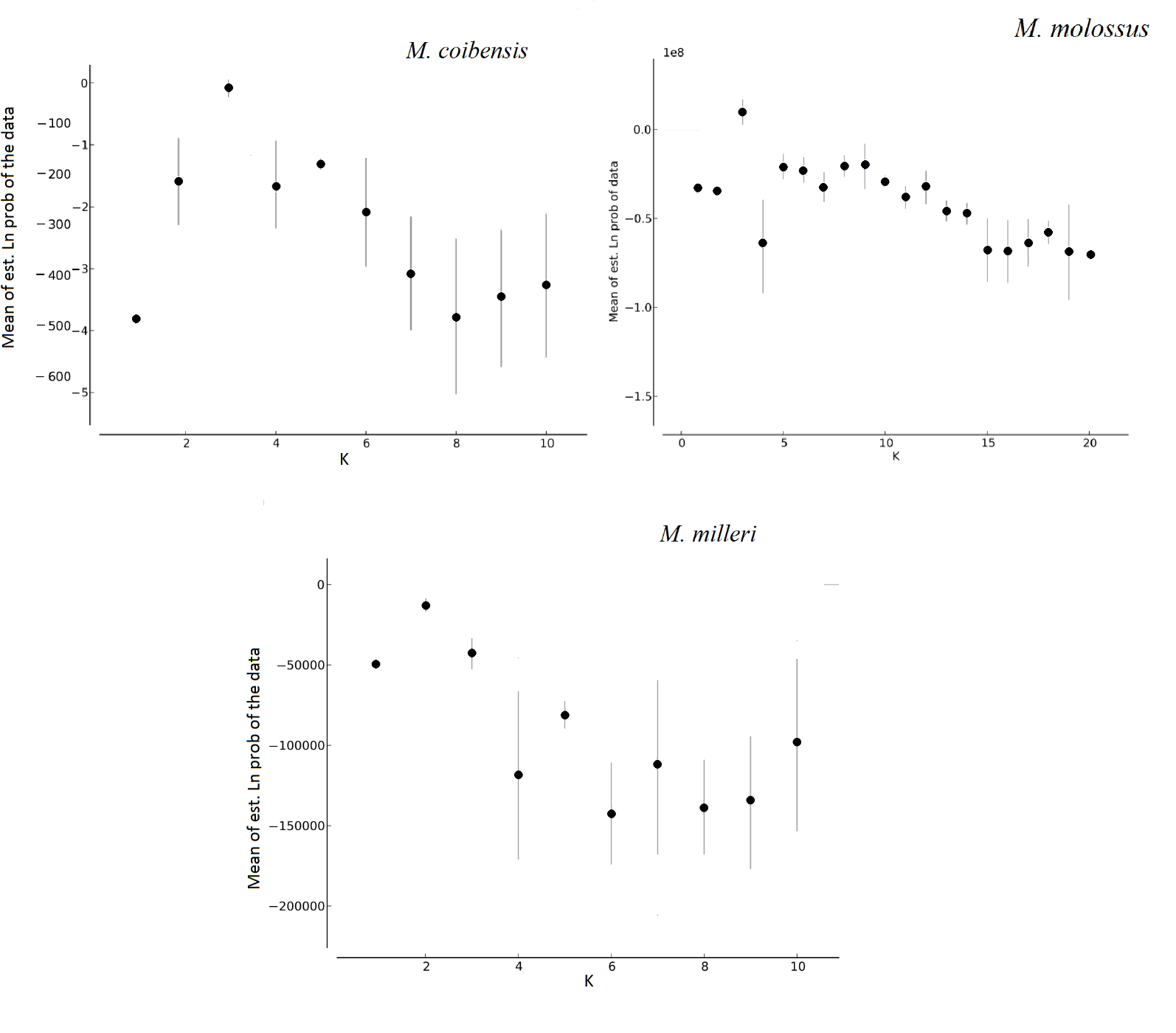


Figure S4 – Structure bar plots for K values above the highest likelihood values. These higher k-values demonstrate no additional sub-structuring within populations beyond the highest likelihood of K. K=4 and K=5 for *M. molossus* and for *M. coibensis* and K=3 and K=4 for *M. milleri*. Each vertical bar along the x-axis represents the genotype of an individual. The y axis indicates the posterior probability of a genotype belonging to one or more clusters.


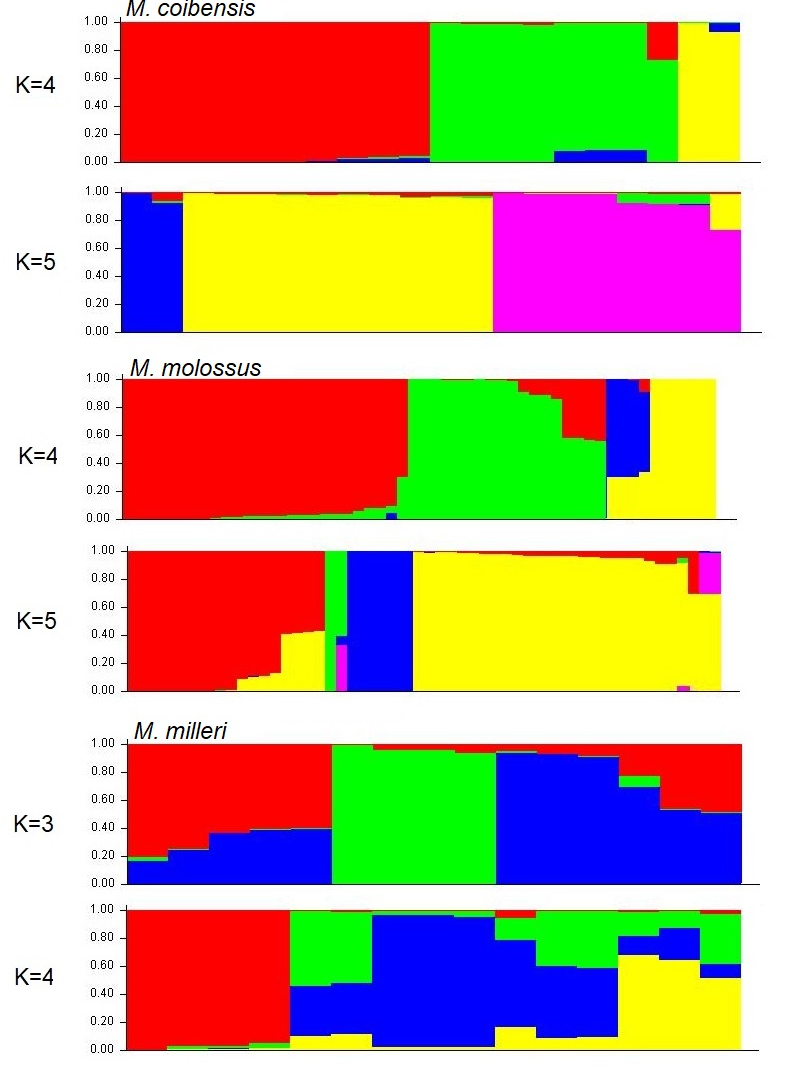


Figure S5 – Principal component analysis (PCA) of pairwise individual genetic distances with 95% confidence ellipses among populations within 3 species of *Molossus*: A- *M. coibensis*, B- *M. milleri*, and C- *M. molossus*.


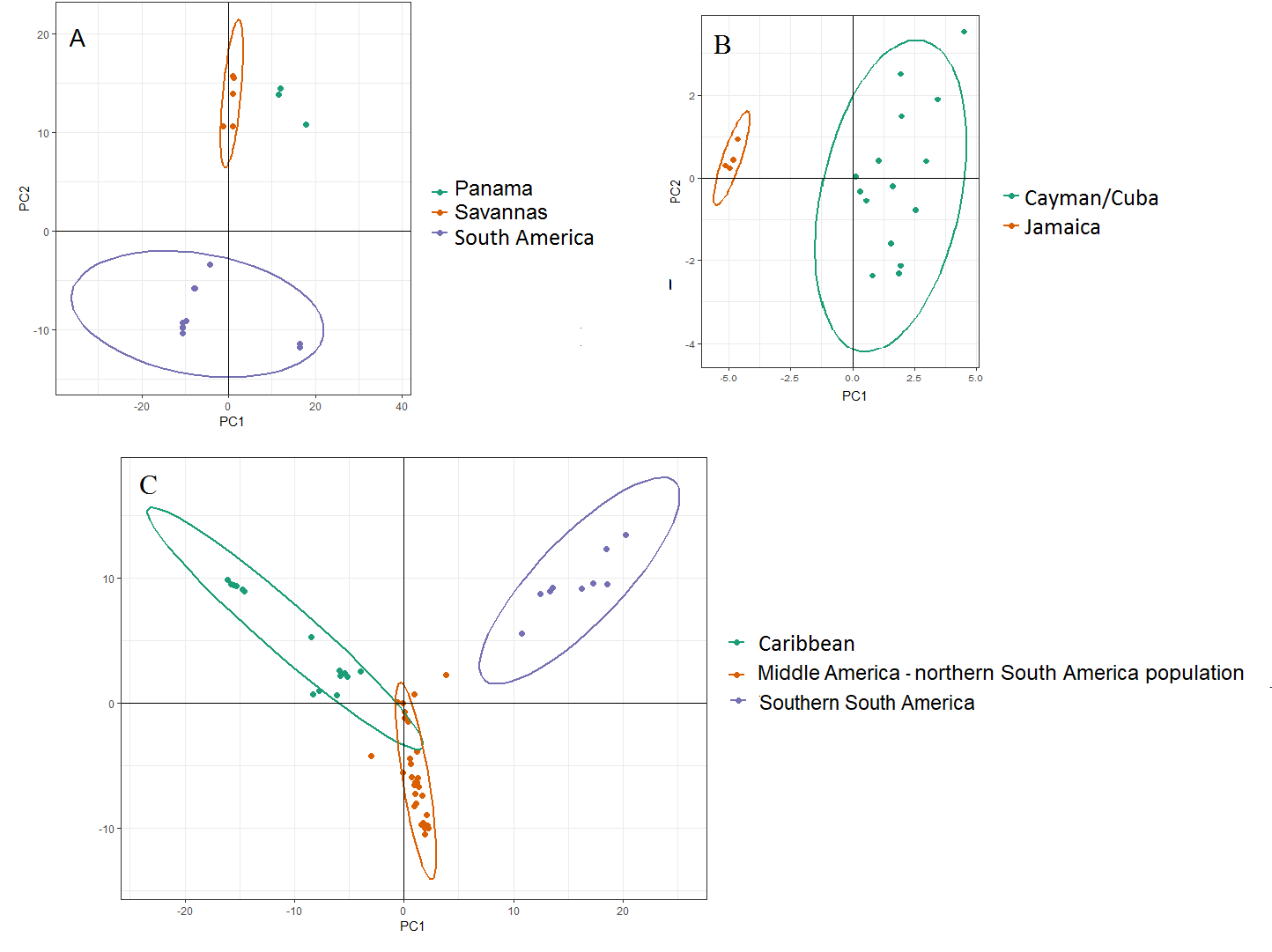


Figure S6 – Percentage of variance explained by the eigenvalues of the Principal Component 
Analysis of pairwise individual genetic distances among populations within 3 species of 
*Molossus*: A- *Molossus coibensis*, B- *M. molossus*, and C- *M. milleri*.


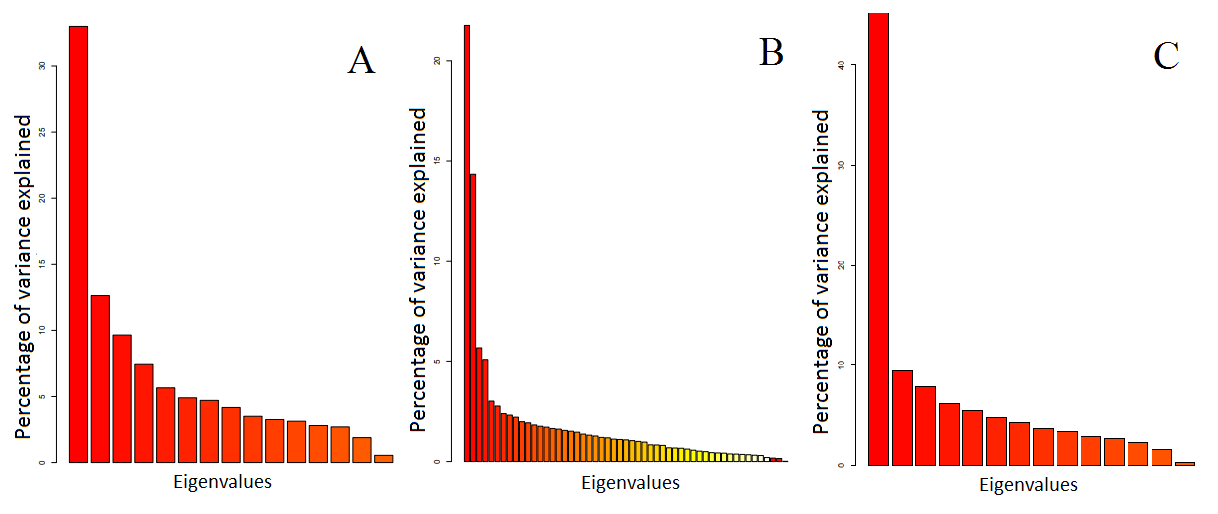


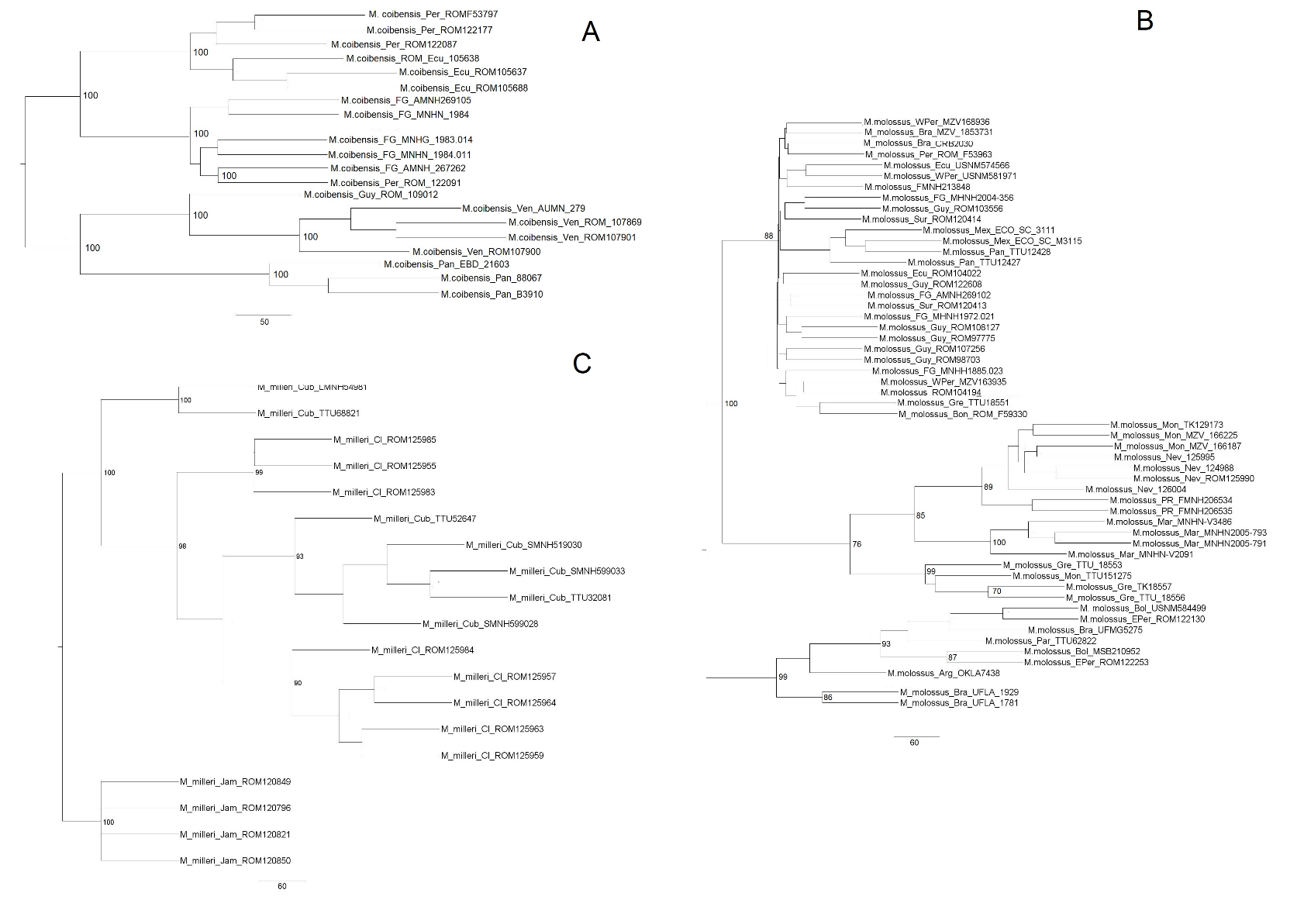


Figure S7 – Coalescence trees for 3 species of *Molossus*: A- *M. coibensis*, B – *M. molossus*, and C- *M. milleri*. Nodes with greater than 70% bootstrap support are indicated.
